# Nuclease Modulates Biofilm Formation in Community-Associated Methicillin-Resistant *Staphylococcus aureus*

**DOI:** 10.1371/journal.pone.0026714

**Published:** 2011-11-11

**Authors:** Megan R. Kiedrowski, Jeffrey S. Kavanaugh, Cheryl L. Malone, Joe M. Mootz, Jovanka M. Voyich, Mark S. Smeltzer, Kenneth W. Bayles, Alexander R. Horswill

**Affiliations:** 1 Department of Microbiology, Roy J. and Lucille A. Carver College of Medicine, University of Iowa, Iowa City, Iowa, United States of America; 2 Department of Veterinary Microbiology, Montana State University, Bozeman, Montana, United States of America; 3 Department of Microbiology and Immunology, University of Arkansas for Medical Sciences, Little Rock, Arkansas, United States of America; 4 Department of Pathology, Nebraska Medical Center, University of Nebraska, Omaha, Nebraska, United States of America; National Institutes of Health, United States of America

## Abstract

Community-associated methicillin-resistant *Staphylococcus aureus* (CA-MRSA) is an emerging contributor to biofilm-related infections. We recently reported that strains lacking sigma factor B (*sigB*) in the USA300 lineage of CA-MRSA are unable to develop a biofilm. Interestingly, when spent media from a USA300 *sigB* mutant was incubated with other *S. aureus* strains, biofilm formation was inhibited. Following fractionation and mass spectrometry analysis, the major anti-biofilm factor identified in the spent media was secreted thermonuclease (Nuc). Considering reports that extracellular DNA (eDNA) is an important component of the biofilm matrix, we investigated the regulation and role of Nuc in USA300. The expression of the *nuc* gene was increased in a *sigB* mutant, repressed by glucose supplementation, and was unaffected by the *agr* quorum-sensing system. A FRET assay for Nuc activity was developed and confirmed the regulatory results. A USA300 *nuc* mutant was constructed and displayed an enhanced biofilm-forming capacity, and the *nuc* mutant also accumulated more high molecular weight eDNA than the WT and regulatory mutant strains. Inactivation of *nuc* in the USA300 *sigB* mutant background partially repaired the *sigB* biofilm-negative phenotype, suggesting that *nuc* expression contributes to the inability of the mutant to form biofilm. To test the generality of the *nuc* mutant biofilm phenotypes, the mutation was introduced into other *S. aureus* genetic backgrounds and similar increases in biofilm formation were observed. Finally, using multiple *S. aureus* strains and regulatory mutants, an inverse correlation between Nuc activity and biofilm formation was demonstrated. Altogether, our findings confirm the important role for eDNA in the *S. aureus* biofilm matrix and indicates Nuc is a regulator of biofilm formation.

## Introduction

*Staphylococcus aureus* is an opportunistic pathogen capable of causing a diverse spectrum of acute and chronic infections. Methicillin-resistant *S. aureus* (MRSA) has received considerable attention due to reports that invasive MRSA infections are surpassing other infectious agents as a cause of death [1]. Over the past decade, the healthcare challenge has worsened with an epidemic wave of MRSA in the community, also called community-associated MRSA or CA-MRSA. These strains are known for causing severe invasive infections not seen in previous epidemic waves of antibiotic resistance [2], [3].

The emergence of CA-MRSA has led to a growing number of reports that these strains are also an important cause of chronic disease, such as infective endocarditis [4], osteomyelitis [5], [6], and foreign body infections [7]. The common theme of these various chronic infections is adherence to a host surface and persistence in the presence of immune defenses and antibacterial therapy. Generally, these types of persistent communities are considered to be growing as biofilms, defined as surface-attached communities of cells encased in an extracellular polymeric matrix that are more resistant to antimicrobial agents.

With a recent surge in studies on *S. aureus* biofilms, our knowledge of the properties of these structured communities continues to develop. One area of recent interest is the matrix material, which displays significant divergence across the Staphylococci. The polysaccharide intercellular adhesin (PIA) is a dominant component of the *Staphylococcus epidermidis* biofilm matrix [8], but there are increasing reports that PIA is less important in the matrix of methicillin-susceptible *S. aureus* (MSSA) and MRSA biofilms [9], [10], [11], [12], [13]. In contrast, many reports have documented a critical role for proteinaceous material in the *S. aureus* matrix [9], [13], [14], [15], [16], [17], [18], [19], [20]. *S. aureus* produces multiple extracellular proteases with self-cleavage activity that can detach cells from surfaces [9], [17], [19], [21], supporting the proposal of a protein-based matrix.

An emerging view of *S. aureus* biofilms is that extracellular DNA (eDNA) has an important structural role in the matrix composition [8], [13], [22], [23]. There is growing appreciation for the contribution of eDNA in a wide range of bacterial biofilms, including *Pseudomonas aeruginosa* [24], [25], [26], *Bacillus* spp. [27], [28], *Haemophilus influenzae* [29], *Neisseria* spp. [30], [31], *Enterococcus faecalis* [32], [33], and *Listeria monocytogenes* [34]. For *S. aureus*, the source of matrix eDNA is thought to be chromosomal DNA released through the controlled lysis of a subpopulation of cells [22], [23]. In an intriguing analysis of the *S. aureus* eDNA composition, Izano *et al.* used a range of restriction enzymes to demonstrate that fragments of at least 11 kb are required to maintain biofilm integrity [8], suggesting the eDNA has to be of sufficient size to serve as effective matrix material.

We have previously shown that *S. aureus* mutants lacking the stress-response alternative sigma factor B (SigB) are unable to form biofilms [12]. Genetic or chemical inhibition of extracellular protease activity restored biofilm capacity [12], [16]. This observation led to our initial hypothesis that the increased protease production in *sigB* mutants, which has also been observed with *sarA* mutants [14], [15], contributed to the biofilm-negative phenotype. In this report, we utilized a biochemical approach to continue our analysis of secreted factors that impact biofilm formation. In contrast to our expectation that a specific protease(s) would be identified, this approach identified secreted nuclease in the spent media of a CA-MRSA *sigB* mutant as a potent anti-biofilm agent. For decades, it has been known that *S. aureus* secretes a thermostable nuclease enzyme, and this activity is highly conserved among clinical isolates and has been used as a marker for direct detection of *S. aureus* in blood cultures [35]. The enzyme is referred to by many different names, such as micrococcal nuclease, thermonuclease, deoxyribonuclease and DNase, and hereafter we will refer to the enzyme as “nuclease” or “Nuc.” Due to its ease of purification [36], the Nuc protein became a favorite among enzymologists and crystallographers, leading to numerous kinetic, protein folding and structural studies [37], [38], [39], [40], [41]. By the time *S. aureus* molecular genetic techniques emerged in the 1980's, interest in Nuc had waned, and less is known about the biological contribution of this enzyme. Herein, we examined the regulation of the *nuc* gene and present a new role for the encoded enzyme in biofilm maturation.

## Results

### Fractionation of LAC spent media identifies secreted nuclease as an anti-biofilm factor

**Figure 1:**
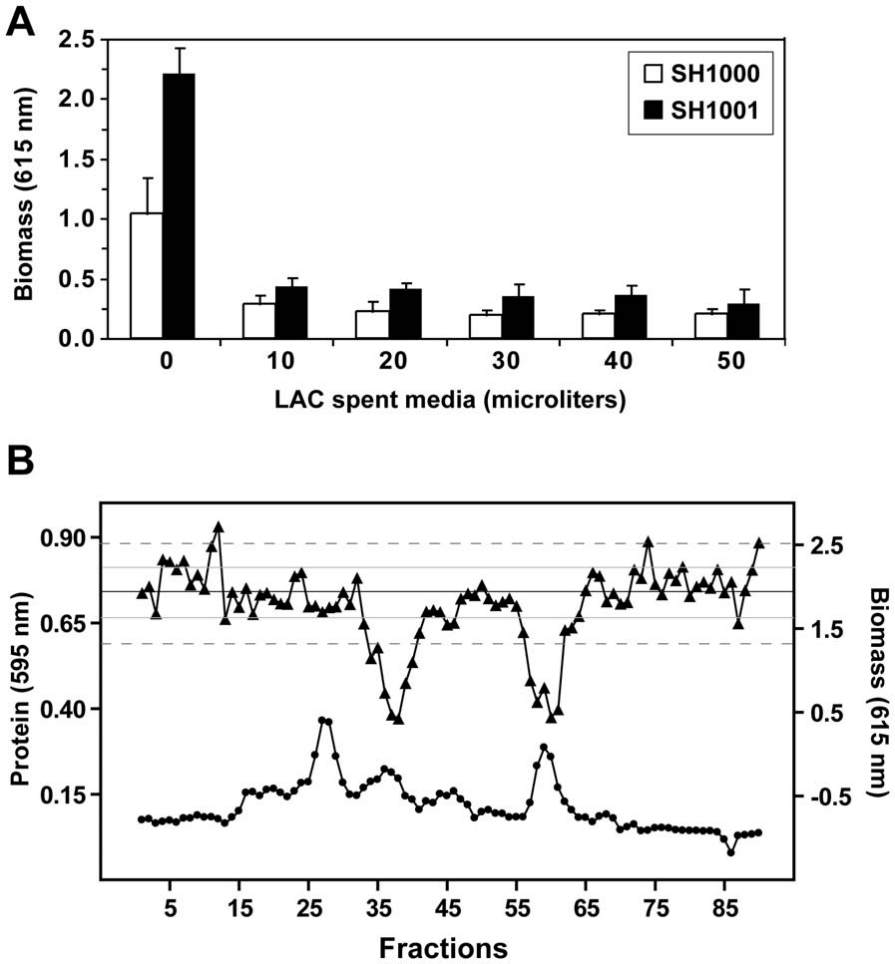
Anti-biofilm activity of spent media from LAC *sigB* mutant. **A.** Cell-free spent media of strain AH1483 (LAC Δ*sigB*) was prepared and varying amounts were incubated with SH1000 (white bars) or SH1001 (black bars) in microtiter biofilm assays. **B.** AH1483 spent media was fractionated on cation exchange resin. Relative protein concentration (closed circles) in the fraction was approximated using Bradford reagent, and each fraction was tested for inhibition of biofilm formation (closed triangles) using SH1001. Horizontal lines display biofilm formation for control wells to which column equilibration buffer was added. The middle black line is the average biofilm formation, and the lines above and below indicate 1 (thin line) and 2 (dashed line) standard deviations from the mean. The two peaks of anti-biofilm activity (fractions 34–40 and 57–61) were more than 2 standard deviations from the mean.

We recently demonstrated that sigma factor B (*sigB*) is essential for biofilm formation in *S. aureus* in both the MSSA strain SH1000 and the CA-MRSA strain “LAC” [12]. Strain LAC is a member of the pulse field gel electrophoresis (PFGE) type “USA300”, which is the dominant clone of the CA-MRSA [42]. Strain LAC has been the subject of extensive transcriptional, proteomic, and *in vivo* pathogenic analysis [43], [44], [45], and we have continued studies on this strain as a model for USA300 chronic infection. The reason for the biofilm negative phenotype of the LAC *sigB* mutant is unknown, but we reasoned that increased levels of extracellular proteases were contributing to this phenotype. To investigate this possibility, we prepared cell-free spent media from LAC *sigB* and discovered that the presence of this spent media inhibited biofilm formation by other wild-type *S. aureus* strains. For this experiment, we used the SigB proficient model strain SH1000 [46], which is a strong biofilm former in microtiter based assays [9], [12] and thus served as a standard to test anti-biofilm activity of spent media. As shown in Figure 1A, as little as 10 µL of spent media from the LAC *sigB* mutant inhibited biofilm formation of SH1000. To rule out the possibility of detachment mediated by autoinducing peptide (AIP) present in the culture supernatant [9], an *agr* mutant (SH1001) unresponsive to AIP was used as the test strain, and the same inhibition of attachment was observed. We concluded that the LAC *sigB* mutant was secreting one or more anti-biofilm factors independent of AIP.

**Figure 2:**
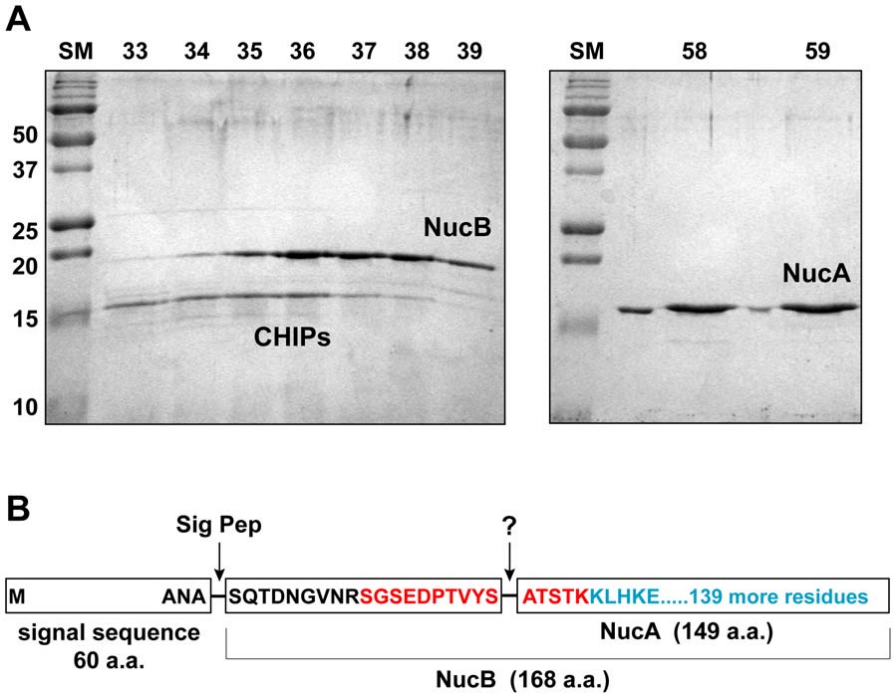
Secreted nuclease (Nuc) is the anti-biofilm factor in LAC *sigB* spent media. **A.** The two major peaks of activity identified in cation-exchange chromatography were separated by SDS-PAGE. Fraction numbers are listed on the top of the gels. The gel on the left shows peak 1 and on the right shows peak 2. Protein bands were excised and identified by MALDI mass spectrometry as NucB and CHIPs in peak 1, and NucA in peak 2. **B.** Schematic of nuclease protein domains. For NucA, only tryptic peptides corresponding to the blue colored residues were identified by MALDI following trypsin digestion. The typtic peptide drawn in red was not observed. For NucB, the blue residues and the additional red colored residues at the amino terminus were identified by MALDI. The indicated location of the cleavage event (between Ser and Ala) that yields the shorter NucA is based on the report of Davis et al. [39].

In an initial attempt to identify the secreted factor, the spent media of the LAC *sigB* mutant was fractionated by anion-exchange chromatography. When column fractions were assayed for their ability to inhibit microtiter plate biofilm formation by SH1001, only the flow-though fractions contained anti-biofilm activity, indicating the unknown factor was cationic. Spent media was fractioned on cation-exchange resin, and two dominant peaks of anti-biofilm activity were identified through microtiter biofilm testing with strain SH1001 (Fig. 1B). These peaks were active beyond two layers of standard deviation in the microtiter biofilm assay. The samples were separated by SDS-PAGE to visualize protein content, and the first active fraction contained two protein bands of ∼14 and 19 kDa, respectively, and the second contained one ∼16 kDa protein (Fig. 2A). Each of these three protein bands was excised from the SDS-PAGE gel, subjected to trypsin digestion, and the peptide fragments were then identified by MALDI mass spectrometry. Following database comparison, the 14 kDa band was identified as chemotaxis inhibitory protein (CHIPS), while the 16 and 19 kDa proteins were identified as different-length forms of the secreted Nuc enzyme (Fig. 2B). Based on the original Nuc naming convention, the longer form of Nuc released by signal peptidase processing is called “NucB” [39]. This protein has a pI of ∼9.3 and elutes from the cation-exchange column first. The shorter processed form of the enzyme called “NucA” has a higher pI of ∼9.5 and elutes later during cation exchange. The processing event removes 19 amino acids from the amino-terminal end and is catalyzed by an unknown protease (Fig. 2B). Considering Nuc was identified in both peaks of anti-biofilm activity, and the processed form of Nuc was pure in the second peak, we concluded that Nuc was the secreted factor in LAC *sigB* mutant spent media that inhibited biofilm attachment. To confirm this finding, purified Nuc enzyme (Worthington Biochemicals) was tested with SH1001 in a microtiter biofilm assay. Similar to testing of the fractionated spent media, Nuc enzyme blocked biofilm formation in a dose-dependent manner (data not shown).

### Regulation of nuclease gene expression

**Figure 3:**
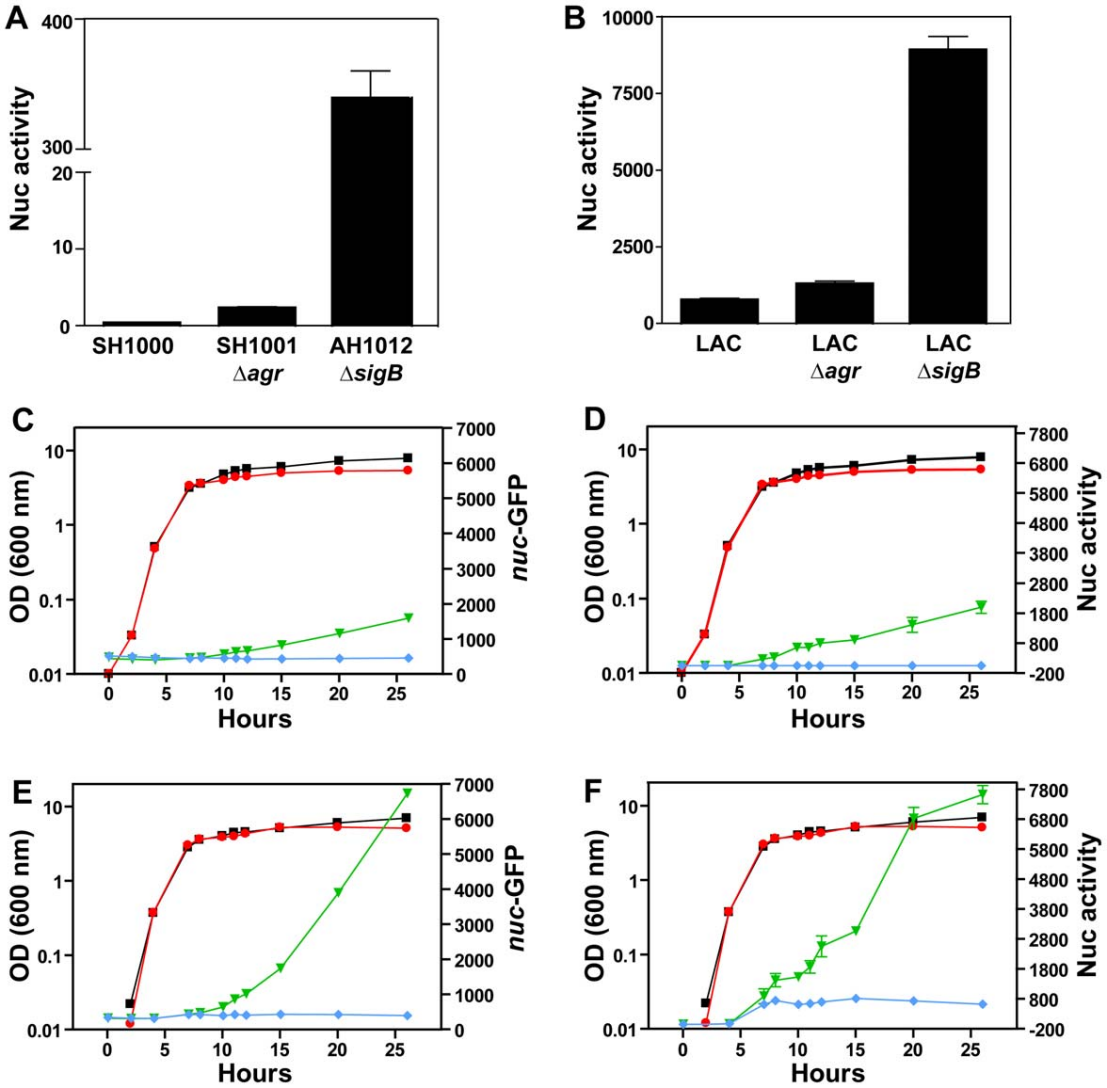
Regulation of Nuc levels in strain LAC. **A.** Extracellular Nuc activity of SH1000 and Δ*agr*::Tet and Δ*sigB* mutants in this genetic background. **B.** Extracellular Nuc activity of LAC and Δ*agr*::Tet and Δ*sigB* mutants in this genetic background. Panels **C–F** represent timecourse results with strain LAC. The P*_nuc_*-sGFP promoter fusion (pCM20) was placed into LAC WT (**C, D**) and Δ*sigB* mutant (**E,F**), and the strains were grown in BHI with and without 0.4% glucose supplementation. Plots of growth versus *nuc* GFP reporter are **C, E**. For labels of these plots, OD of growth in BHI (black closed circles), OD of growth in BHI + glucose (red circles), *nuc* reporter in BHI (green triangles), *nuc* reporter in BHI + glucose (blue diamonds). Plots of growth versus Nuc activity are **D, F**. The Nuc activity labels correlate with color coding of *nuc* reporter measurements, and the OD plots are the same.

Knowing that the Nuc enzyme could inhibit biofilm development, we hypothesized that *S. aureus* regulates this enzyme in order to control eDNA levels for biofilm maturation. To evaluate Nuc regulation, we developed an enzyme activity based on fluorescence resonance energy transfer (FRET) (see Methods and Materials for additional details). The assay was verified using purified Nuc enzyme, and as anticipated, the assay responded in a dose-dependent manner to Nuc (data not shown). Purified enzyme used to generate a standard curve for calculation of activity units throughout this report. Nuc enzyme activity levels were measured in *sigB* and *agr* global regulatory mutants in the SH1000 and LAC genetic backgrounds (Fig. 3A & 3B). In *sigB* mutants, Nuc levels were up ∼240-fold in SH1000 and 11.5-fold in LAC. Contrary to our expectations, inactivation of *agr* had no adverse effect on Nuc activity levels.

To expand on these regulatory studies, time courses were performed in LAC WT and regulatory mutants using media with and without glucose supplementation. Addition of glucose to growth media is a common practice used to simulate biofilm forming conditions for *S. aureus*, and cells grown in media without supplemental glucose do not form biofilms [9]. Throughout the time course, transcription of the *nuc* gene was monitored with a promoter fusion to sGFP (plasmid pCM20), Nuc activity levels were measured with the FRET-based assay, and the pH of the media was determined. As anticipated from published reports [47], the pH dropped during logarithmic growth as the glucose was consumed and acidic metabolites were excreted, and no significant difference was observed in the pH profiles of any of the strains tested (data not shown). In the presence of excess glucose, the pH dropped to ∼5 for each strain, and did not recover to a neutral pH in the time frame of the experiment (data not shown). Like many extracellular enzymes, expression of *nuc* correlated with growth. Transcription of *nuc* in the WT strain, as measured by the P*_nuc_*-GFP reporter, was first detected during logarithmic growth and continued into early stationary phase (Fig. 3C). Likewise, extracellular Nuc enzyme, as measured using the FRET activity assay, was first detected during logarithmic growth and continued to accumulate into earlier stationary (Fig. 3D), reaching 1955 U/ml at the end of the time course. Supplementation of the media with 0.4% glucose repressed *nuc* transcription (Fig. 3C) and prevented Nuc enzyme from accumulating (Fig. 3D), although trace activity (0.2–0.5 Units/ml) was detected during early logarithmic growth before dropping to undetectable levels. In the absence of supplemental glucose, the timing of *nuc* transcription and accumulation of Nuc enzyme by the *sigB* mutant was similar to that of WT, except the *sigB* mutant accumulated several fold more transcript (Fig. 3E) and Nuc enzyme (Fig. 3F), reaching 7670 U/ml at the end of the time course. The effect of 0.4% supplemental glucose on *nuc* expression was significantly attenuated in the *sigB* mutant. Nuc activity levels were still quite high, reaching 660 U/ml in late logarithmic growth and remaining in the 660–860 U/mL range through the end of the time course (Fig. 3FD). In contrast, and in support of the Nuc activity assays in Fig. 3B, *nuc* expression and enzyme activity profiles of the *agr* mutant were identical to those of WT (data not shown).

### Controlled nuc gene expression modulates biofilm formation

**Figure 4:**
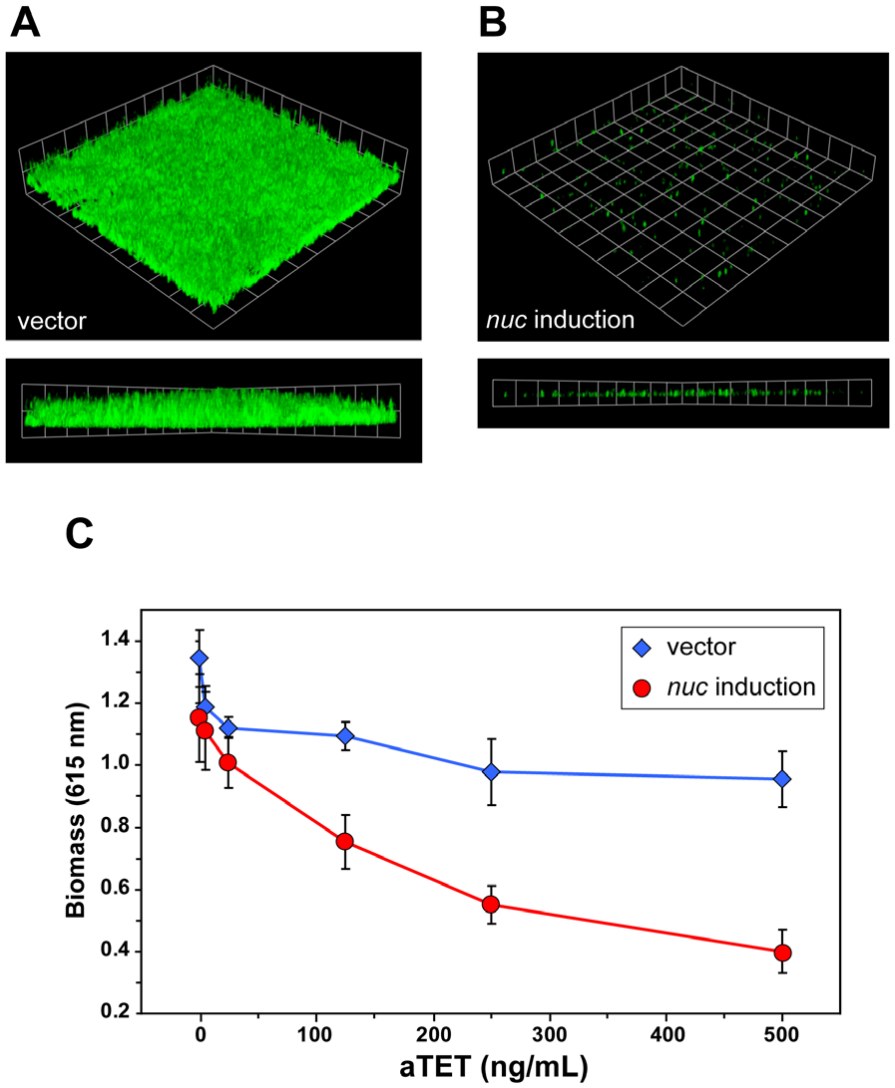
The effect of controlled *nuc* gene expression in biofilms. For images **A** and **B**, biofilms were grown in TSB supplemented with 0.2% glucose and 100 ng/mL aTet and post-stained with Syto9. The *nuc* gene was expressed from an aTet-inducible promoter in plasmid pALC2073. A *z* series of images were obtained with CLSM and reconstructed for the 3D rendering. Beneath each 3D reconstruction is a XZ cross-section of the biofilm with each side of a grid square at 20 µm. **A.** SH1001 with pALC2073 (vector). **B.** SH1001 with pALC2073-*nuc* (*nuc* induction). For a dose-response test (**C**), SH1001 with pRMC2 (vector) was compared to SH1001 with pRMC2-*nuc* (*nuc* induction) comparison in microtiter biofilm assays at increasing aTet concentrations.

Considering that Nuc levels are repressed during growth in biofilm media, we hypothesized that controlled *nuc* expression could impact biofilm formation. To investigated this hypothesis we transformed tetracycline-inducible expression vectors containing *nuc* into SH1001, a strain that normally produces low levels of Nuc (Fig. 3A) and is a good biofilm former (Fig. 1A), and we tested whether induction of *nuc* expression could disrupt biofilm formation in flow cells and microtiter plates. In a flow cell experiment with anhydrotetracycline (aTet) induction, the strain harboring pALC2073-*nuc* failed to develop biofilm (Figure 4B), whereas the strain harboring empty vector established a robust biofilm (Figure 4A). Similarly in a microtiter plate biofilm assay, SH1001 containing pRMC2-*nuc* displayed a dose-dependent decrease in biomass as the concentration aTet inducer was increased, whereas SH1001 containing empty vector did not (Fig. 4C). As anticipated, Nuc activity levels rose in conjunction with the increased levels of aTet inducer (data not shown). Coupled with the time course studies in Figure 3, it is evident that glucose suppression of Nuc levels enhances biofilm formation and distorting this regulatory event has a negative impact on biofilms.

### Construction and characterization of a LAC nuc mutant

**Figure 5:**
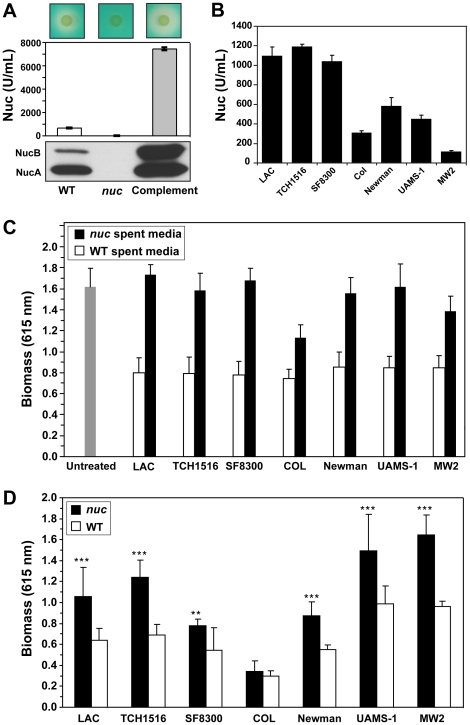
Characterization of MSSA and MRSA *nuc* mutants. **A.** LAC WT, *nuc* mutant, and complemented strains were tested in three conditions: DNase agar plates (top panels) with zones of clearing indicating secreted Nuc activity, Nuc enzyme activity measurement (middle panel), and immunoblot for Nuc protein (bottom panel). The long form (NucB) and processed short form (NucA) are indicated. **B.** Extracellular Nuc activity of various WT *S. aureus* strains. **C.** Effect of *nuc* mutant (black bars) and WT (white bars) spent media on inhibition of SH1001 biofilm formation. Values are the average of 16 wells. Untreated control (n = 36 wells) is shown on the left as a gray bar. **D.** Biofilm formation of *nuc* mutants (black bars) versus WT strains (white bars). Values are the average of 8 wells.

With an important role for Nuc in biofilm modulation being revealed, we assessed the contribution of Nuc to biofilm formation in strain LAC. A *nuc* mutant (AH1680) was constructed and characterized using multiple different methods to assess Nuc function. Using DNA agar plates, the secreted DNase activity was markedly lower in the *nuc* mutant and restored in a complemented strain (Fig. 5A). Nuc activity measured with the FRET assay was almost undetectable in the mutant and overproduced ∼10-fold in the complemented strain (Fig. 5A). Using Nuc antibodies, secreted Nuc protein was absent from the mutant and somewhat overproduced in the complemented strain (Fig. 5A). The long (NucB) and short (NucA) forms of nuclease were apparent in the immunoblot for both WT and the complemented mutant strain, while both bands were absent in the *nuc* mutant. As anticipated, both long and short forms of Nuc are active as confirmed by DNA zymography (Fig. S1A & S1B) and the FRET-based enzyme assay (data not shown). To examine the processing of NucB to NucA, the LAC *sigB* mutant was grown with protease inhibitors E64, EGTA, and PMSF to inhibit the cysteine, metallo, and serine classes of proteases. In an immunoblot (Supplementary Fig. 1C), the longer NucB form of the enzyme was restored with E64, suggesting a cysteine protease is contributing to the cleavage event, and to a lesser extent, calcium chelation with EGTA also inhibited the processing. Finally, exoprotein analysis by SDS-PAGE indicated the only difference between the extracellular proteomes of the WT, complemented and *nuc* mutant strains was the absence of the Nuc protein bands in the mutant (data not shown).

### Characterization of nuc mutants in other strain backgrounds

To expand the Nuc studies beyond the LAC genetic background, spent media from LAC and additional strains were assayed for Nuc activity using the FRET-based assay and for the ability to prevent biofilm formation using a SH1001 microtiter assay. The strains tested were MSSA strains Newman [48] and UAMS-1 [49]; hospital-associated MRSA isolate COL [50]; and CA-MRSA isolates MW2 [51], SF8300 [52], and TCH1516 [53]. Some variability in Nuc activity was observed in the spent media from cultures grown in TSB (Fig. 5B). The USA300 strains had the highest Nuc levels, with LAC, SF8300 and TCH1516 all accumulating approximately 1100 U/ml of activity. The Nuc levels were 310 U/ml in COL, 585 U/ml in Newman, and 452 U/ml in UAMS-1, all substantially lower than the USA300 strains. MW2 had the lowest amount of Nuc (116 U/ml), accumulating only 10% of the USA300 activity. The significantly longer doubling times of Newman (46 min) and COL (64 min), relative to that of the USA300 strains (32 min) [54], may account for the lower Nuc accumulation in these strains. In contrast, the low level in MW2 is indicative of altered growth-dependent expression of *nuc*, since this strain has a doubling time similar to USA300 strains [51]. The spent media from all the strains was able to prevent biofilm formation by SH1001 (Fig. 5C), with high statistical significance compared to the untreated control. Considering only ∼12 U/ml Nuc is present in the MW2 spent media sample following dilution, the amount of Nuc needed to prevent SH1001 microtiter biofilm formation is less than this amount.

To determine whether Nuc was contributing to the anti-biofilm activity of spent media, *nuc* mutations were generated in each of the strains assayed in Fig. 5B. After confirming the mutations through molecular analysis, DNase agar plate assays, and FRET-based activity assays (data not shown), the ability of spent media from *nuc* mutant cultures to prevent biofilm formation by SH1001 was assayed. In all cases, when compared to the corresponding WT control, the spent media from the *nuc* mutant had reduced ability to prevent SH1001 biofilm formation (Fig. 5C). For the USA300 (LAC, SF8300, TCH1516), Newman and UAMS- 1 strains, the amount of SH1001 biofilm formed in the presence of *nuc* mutant spent media was statistically indistinguishable from the untreated control, implying that Nuc could account for the majority of the anti-biofilm activity observed with this assay. In the presence of spent media from either the COL or MW2 *nuc* mutants, the amount of SH1001 biofilm formed was more than the corresponding WT media, but less than the untreated control.

To determine whether the *nuc* mutants share a common biofilm deficient phenotype, microtiter plate assays were performed for each WT strain and its *nuc* mutant counterpart (Fig. 5D). In all but one case, the introduction of a *nuc* mutation resulted in a significant enhancement of biomass accumulation. The only exception to the enhancement phenotype was strain COL, which was the weakest biofilm former of the group. The COL phenotype could be due in part to the additional anti-biofilm activity that was revealed by the spent media SH1001 microtiter plate assay (Fig. 5C).

### Flow cell biofilms of the LAC nuc mutant

**Figure 6:**
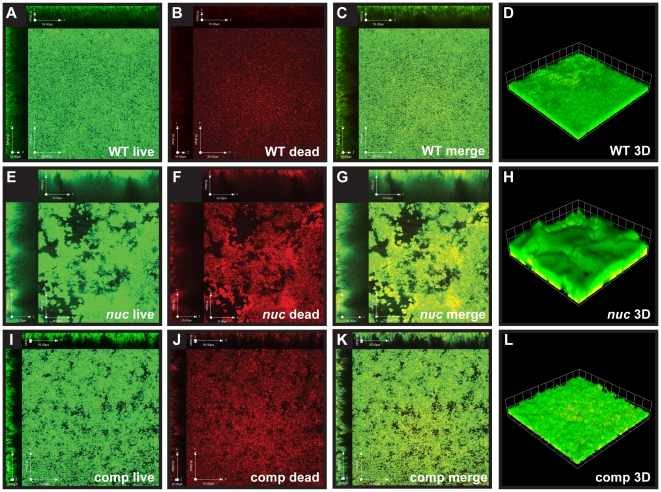
Growth of the LAC *nuc* mutant in a flow cell. The LAC WT (**A–D**), *nuc* mutant (**E–H**), and complemented (**I–L**) strains were grown in flow cell biofilm conditions for 3 days. The biofilms were post-stained with Syto9 (live stain, green) and Toto3 (dead stain, red). Top-down and cross-section CLSM images were taken for Syto9 (**A, E, I**) and Toto3 (**B, F, J**) channels. A merged image of these two channels was also prepared (**C, G, K**). Finally, a *z* series of merged images was obtained for a 3D reconstruction of the biofilm (**D, H, L**) and each side of a grid square is 20 µm.

We recently reported that a *nuc* mutation in the MSSA strain UAMS-1 increases the biofilm forming capacity in a flow cell [22]. To learn whether CA-MRSA displays a similar phenotype, the LAC WT, *nuc* mutant and complemented strains were grown in flow cells, and biofilms were post-stained with the live cell stain Syto9 and dead stain Toto3 to indicate eDNA and dead cells (Fig. 6). The Toto3 dye was selected because its far-red emission collection does not overlap with that of Syto9, and Toto3 has increased sensitivity as compared to another common dead stain, propidium iodide, in detecting eDNA [22]. Following confocal fluorescence microscopy, we observed that average thickness of biofilms was markedly increased in the *nuc* mutant (38.5 µm vrs. 18.1 µm for WT), and the WT phenotype was restored with complementation of *nuc* on a plasmid (14.7 µm). We also observed an increase in Toto-3 staining near the substratum of the *nuc* mutant biofilm. This increase in Toto-3 staining could be indicative of more eDNA at the initial point of bacterial attachment, but this staining technique does not distinguish between eDNA and dead cells.

### High molecular weight eDNA accumulates in culture media of the nuc mutant

**Figure 7:**
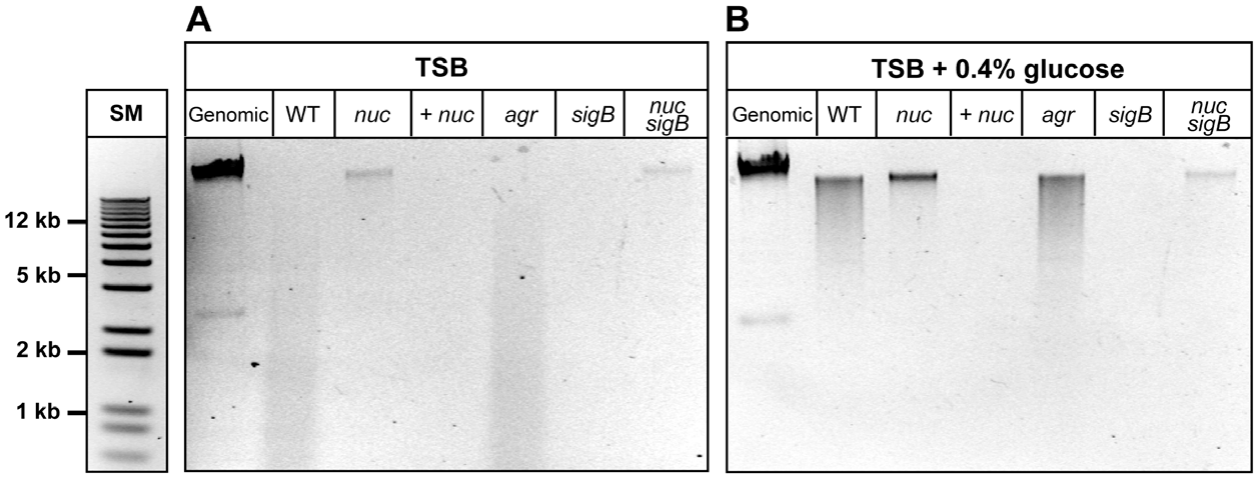
Accumulation of high MW eDNA in the *nuc* mutant. The eDNA was purified from the spent media of LAC WT and strains with mutations in the *nuc*, *agr*, and *sigB* loci. A *nuc* complement (+ *nuc*) and *nuc sigB* double mutant was also included. **A.** Agarose gel of eDNA purified from strains grown in TSB. **B.** Agarose gel of eDNA purified from strains grown in TSB with 0.4% glucose supplementation. In each gel, lane “SM” is a DNA size marker with a top size of 12 kb. Purified chromosomal DNA was included as a control and is shown in the lane labeled “genomic”.

Although expression of *nuc* is repressed under biofilm forming conditions (Fig. 3), the LAC WT biofilm is still substantially reduced when compared to the *nuc* mutant in microtiter (Fig. 5) and flow-cell experiments (Fig. 6). We hypothesized that the *nuc* mutant would accumulate eDNA, while residual Nuc activity in the WT strain would degrade eDNA into smaller fragments, negatively impacting biofilm formation. To assess eDNA accumulation and fragment size, eDNA was isolated from media of cultures grown with and without glucose supplementation and separated in agarose gels (Fig. 7). Without excess glucose, eDNA isolated from WT and an *agr* mutant strain appears as a smear of lower molecular weight (MW) DNA fragments (Fig. 7A). The intensity of these smears is highest at the bottom of the gel under 5 kb in size, indicating that the majority of eDNA in these lanes is low MW. In contrast, eDNA isolated from the *nuc* mutant is similar in size to purified, high MW genomic DNA with minimal degradation, and this phenotype can be complemented. In the *sigB* mutant where Nuc levels are high, the eDNA is completed degraded, and this phenotype can be restored by introducing a *nuc* mutation.

With excess glucose, a high MW band of eDNA is again observed for the *nuc* mutant and the *nuc sigB* double mutant (Fig. 7B). Interestingly, WT and the *agr* mutant have increased amounts of high MW eDNA compared to the low glucose condition (Fig. 7B vrs. 7A), although some degradation was still apparent, which consistent with trace Nuc activity accumulating in the high glucose media. The eDNA in the *sigB* mutant and the complemented *nuc* strain is degraded, presumably due to the high levels of Nuc enzyme in both conditions. Taken together, these experimental observations indicate that the *nuc* mutant accumulates more high MW eDNA compared to WT and a *sigB* mutant, and the introduction of the *nuc* mutation is dominant for this phenotype in variant genetic backgrounds.

### Removal of nuclease improves sigB mutant biofilms

**Figure 8:**
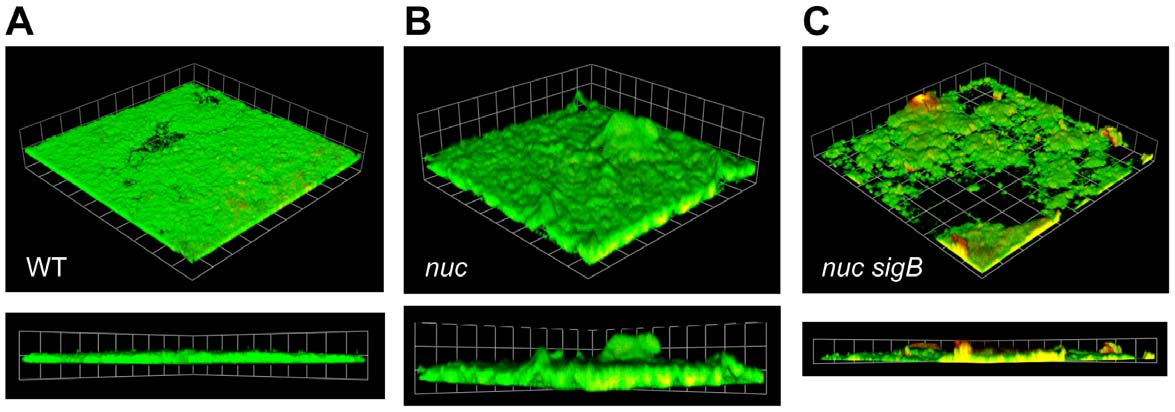
Rescuing LAC *sigB* mutant biofilms with *nuc* mutation. The LAC WT (**A**), *nuc* mutant (**B**), and *nuc sigB* double mutant (**C**) strains were grown in flow cell biofilm conditions for 2 days. The biofilms were post-stained with Syto9 (live stain, green) and ToPro3 (dead stain, red). Representative 3D reconstruction and cross-section images from CLSM are shown. Each side of a grid square is 38 µm.

With the apparent correlation of high Nuc levels to a biofilm-negative phenotype, we hypothesized that inactivation of the *nuc* gene in the *sigB* mutant background would restore biofilm-forming capacity. The *nuc sigB* double mutant was constructed in the LAC strain, and biofilm formation was compared to WT and single mutants in *nuc* and *sigB* using flow cells. The controls behaved as anticipated with WT forming a biofilm (Fig. 8A) and the *nuc* mutant showing increased biomass accumulation (Fig. 8B), while the *sigB* mutant formed no biofilm (data not shown), which is consistent with our previous report that a *sigB* mutant is incapable of forming a biofilm under flow-cell conditions [12]. In contrast, the *nuc sigB* double mutant (Fig. 8C) formed a patchy biofilm compared to WT and the *nuc* mutant. Based on COMSTAT analysis, the average biomass of the *nuc sigB* double mutant (11.1 µm^3^/µm^2^) was similar to WT (11.8 µm^3^/µm^2^), while the average thickness was much reduced (4.3 µm vrs. 16.7 µm), consistent with the uneven distribution of biofilm across the surface. The ability of a *nuc* mutation to partially restore biofilm formation in the *sigB* strain confirms that Nuc activity negatively affects this phenotype. However, eliminating Nuc did not fully restore *sigB* biofilm to a WT level, indicating that other factors also contribute to the *sigB* phenotype.

### Nuc vrs. biofilm correlation

**Figure 9:**
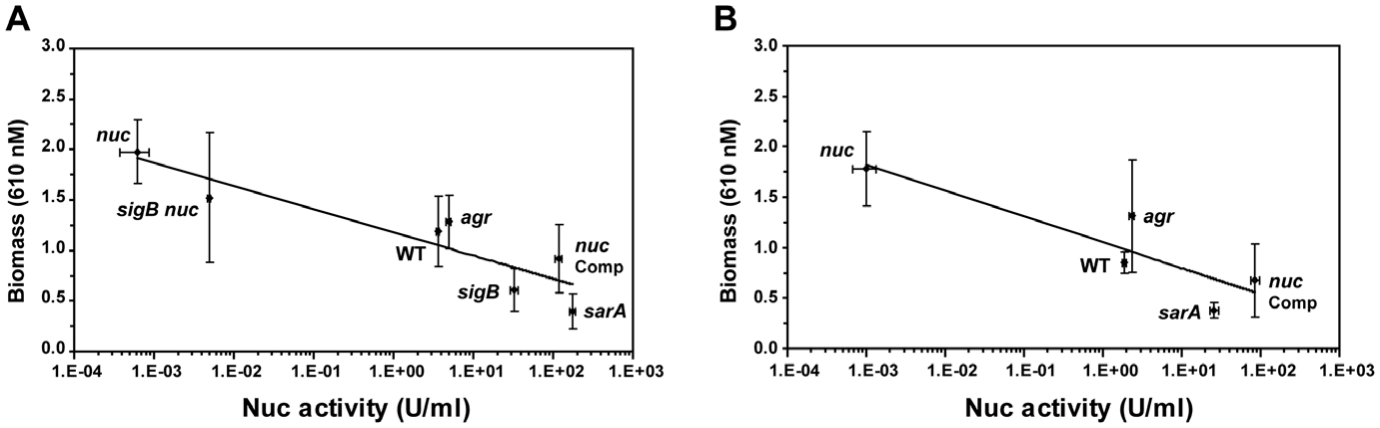
Correlation of Nuc activity with biofilm formation. Nuc activity of mutants in the LAC (**A**) and UAMS-1 (**B**) strain backgrounds was measured and compared to the amount of biomass accumulated by each strain. Nuc activity was measured using a FRET assay and is displayed in U/mL. Biofilm formation was assessed by measuring biomass accumulation in microtiter assays. Logarithmic fits were performed (shown as line on plots) with similar results for each plot.

In order to quantify the inverse relationship between Nuc activity and biomass, microtiter plate biofilm assays were conducted on strains containing mutations that modulate Nuc activity in the LAC WT (Fig. 9A) and UAMS-1 (Fig. 9B) genetic backgrounds. Nuc activity measurements were performed on the filter-sterilized media removed from the microtiter plate wells following growth, allowing for a direct comparison between Nuc activity and biomass. The most notable finding is that the logarithmic fits of the data demonstrate a quantitative relationship between biomass and Nuc activity in the LAC WT and UAMS-1 backgrounds, and these fits are strikingly similar across genetic backgrounds (Fig. 9). Several other observations are consistent with the overall findings in this work: (1) *nuc* mutants accumulated the most biomass, had the lowest measurable Nuc activity, and complementation restored the WT phenotype; (2) a *sigB* mutant had high Nuc activity and accumulated low levels of biomass, and introduction of the *nuc* mutation restored the WT phenotype; (3) *agr* mutants were similar to wildtype strains in both Nuc activity levels and biomass; and (4) *sarA* regulatory mutants had high Nuc activity and accumulated the least amount of biomass. Based on these correlation plots, Nuc activity levels are a strong predictor of biofilm formation across multiple *S. aureus* strain lineages.

## Discussion

We observed that USA300 LAC *sigB* mutant spent media contained potent anti-biofilm activity that counteracted the ability of other wild-type *S. aureus* strains to develop biofilms. In this work, we identified the source of this activity as the secreted Nuc enzyme and further examined the regulation and role of Nuc in biofilm maturation. The discovery of Nuc in the fractionation experiment, and not proteases, was somewhat unexpected. Based on the recent reports that proteases can disperse *S. aureus* biofilms [9], [17], [19], we anticipated identifying proteolytic activity in the fractions that blocked biofilm formation. Multiple reports have demonstrated that overproduction or exogenous addition of SspA (V8) protease cleaves fibronectin binding proteins, which are important in the accumulation of biofilm biomass [19], [21]. In particular, exogenous addition SspA was found to prevent biofilm formation for various strain lineages in a microtiter plate assay that uses uncoated plates and glucose supplementation [19]. Given the similarities between the microtiter assay employed in this report and the published SspA work, we anticipated finding anti-biofilm activity in the spent media associated with this protease. However, we failed to identity SspA (pI = 4.6) in any of the elution fractions from anion-exchange chromatography or flow-through fractions from cation-exchange chromatography.

Several factors may have contributed to lack of proteases in the anti-biofilm fractions. First, it is possible that the concentration of SspA (or other proteases) in the column fractions was too low to prevent biofilm formation. Testing whether this is the case would require a quantitative determination, similar to what we have reported here for Nuc, of the minimal amount of exogenous SspA (or other protease) required to prevent biofilm formation. Second, a number of studies suggest that effects of proteases on biofilm formation require the combined activities of multiple different enzymes. Introduction of single protease mutations into the SH1000 *sigB* background [12], [17] or into the UAMS-1 *sarA* background [15] failed to restore biofilm forming capacity to the *sigB* mutant and *sarA* mutants, respectively. However, simultaneous introduction of the *aur* and *splABCDEF* mutations restored biofilm forming capacity to the SH1000 *sigB* mutant [12], and the addition of protease inhibitor cocktails restored biofilm-forming capacity to the UAMS-1 *sarA* mutant [15]. Given these observations, the failure of the fractionation experiment to establish a link between proteases and anti-biofilm activity is consistent with reports that the coordinated function of multiple enzymes might be required. Lastly, it may be that under altered biofilm assay conditions, such as coating a surface with matrix proteins, SspA or other proteolytic enzymes would have more impact on biofilm formation.

With a newly identified role for Nuc as a biofilm inhibitor, we hypothesized that *S. aureus* must repress enzyme production during biofilm formation. Regulatory studies using a promoter fusion confirmed that the *nuc* gene was repressed by glucose supplementation, which is a common additive used by researchers to induce biofilm formation [9], [10], [19], [55]. As anticipated, mutations in *sigB* lead to increased *nuc* expression and Nuc activity (Fig. 3), confirming microarray studies on these mutants [56], [57]. The reason for the enhanced Nuc levels is unknown but could provide future insights on *nuc* gene regulation. There has long been speculation that Nuc, like other extracellular enzymes, is part of the *agr* regulon [58], [59]. Surprisingly, using both transcriptional fusions and Nuc activity assays, we were unable to find a role for *agr* in Nuc regulation at either the transcriptional or post-transcriptional level. We are currently following up on these observations to further understand Nuc regulation.

Considering that *nuc* is already repressed with glucose supplementation, the question also remained as to why the *nuc* mutant grew a thicker flow cell biofilm than the WT strain (Fig. 6). It is important to note that even with glucose supplementation, trace levels of Nuc activity (0.2–0.5 U/ml) were still detected in the WT spent media. This Nuc activity difference is apparent when looking at the eDNA that accumulates outside the cell. Higher MW DNA accumulated in the *nuc* mutant (Fig. 7), and the length of the eDNA isolated was predominantly over 10 Kb, in range of the previously identified cutoff necessary to maintain intact biofilms [8]. The trace Nuc activity in the WT media may also explain the difference in biomass accumulation in WT vrs. *nuc* mutants in the microtiter biofilm assay (Fig. 5D). We hypothesize that enough high MW eDNA accumulates after 4 hours of growth to allow for biofilm formation to occur. After this threshold point is reached, further cell lysis is not required, perhaps explaining why inhibition of lysis is most effective at the initial growth stages in preventing biofilm formation [22]. Other aspects of the eDNA isolation paralleled Nuc regulation studies, such as when Nuc levels were high in a *sigB* mutant or in media without glucose, the eDNA was digested into smaller fragments. Overall, these findings confirm previous reports and demonstrate that eDNA must be high MW in order to be an effective matrix material [8].

The information acquired on the control of *nuc* gene expression may provide insight on the biofilm phenotypes observed for various *S. aureus* strains and regulatory mutants. Our findings demonstrate that Nuc levels are tightly correlated with biofilm formation, and this correlation applies across different WT stains (Fig. 5). The only strain that behaved differently was COL, which failed to develop a biofilm in the microtiter assay. Further, the spent media from a COL *nuc* mutant still retained anti-biofilm activity (Fig. 5C). Whether this strain produces an additional anti-biofilm factor, such as an extracellular protease or a second nuclease, is not clear. Most *S. aureus* strains tested produced high levels of Nuc, in particular USA300 isolates (Fig. 5B), which might be a factor in the technical challenges of assaying for biofilm formation using uncoated microtiter plates. Our findings also suggest that conditions or mutations leading to distorted Nuc regulation contribute to biofilm phenotypes (Fig. 9). For instance, *sigB* mutants overproduce Nuc even in the presence of glucose suppression (Fig. 3F), leading to the degradation of eDNA (Fig. 7B) and the inability to form biofilms. We demonstrate that removal of Nuc repairs these phenotypes and recovers biofilm capacity (Fig. 7B and 8C), although not completely. It is already appreciated that proteases are an important contributor to the biofilm-negative phenotype of *sigB* and *sarA* mutants [12], [14], [15], and the high level of extracellular proteases likely explains the inability of *sigB nuc* mutants to form a wildtype level of biofilm (Fig. 8C). This proposal would be consistent with our previous observations that the reduction or elimination of protease activity partially restored *sigB* mutant biofilms [12]. Taken together, we conclude that *S. aureus* biofilm formation is simultaneously impacted by high levels of Nuc and extracellular proteases. However, *in vivo* confirmation of this proposal is not yet available. Ongoing studies in our laboratories will begin to address these questions.

Considering our observations and other published reports [8], [22], [23], the release and utilization of eDNA as a matrix material may be an underappreciated factor in the survival of *S. aureus*. There is evidence in previous reports that eDNA is necessary for *S. aureus in vivo* biofilm formation [23], [60]. Although it has been proposed that medical devices become coated with host matrix proteins following implantation [61], [62], [63], a recent report demonstrated that abolition of nuclease production partially restores biofilm formation in a *S. aureus sarA* mutant even in the presence of plasma protein and protease inhibitors [15], suggesting there is potential for eDNA during *in vivo* chronic infection. There are also a growing number of examples where eDNA is required for *in vivo* biofilm formation by other bacterial pathogens [29], [64]. While we have investigated the contribution of *S. aureus* Nuc to modulating eDNA levels, the host nucleases could also be a significant factor *in vivo*. DNase I is the most extensively distributed host nuclease and the enzyme responsible for the majority of DNase activity in blood [65]. In serum samples, reports of DNaseI levels range from 65±27 U/g protein [66], which corresponds to 0.0044±0.0018 U/ml, to higher estimates of 0.356±0.410 U/ml [66] and 2.47±2.48 U/ml [67]. Given these low levels of activity, it is not clear whether there are sufficient quantities of DNaseI to impact *S. aureus* biofilm formation during infection. Clearly, more in-depth studies on the role of eDNA and nuclease enzymes *in vivo* are warranted.

Herein, we have presented evidence that Nuc levels have a significant impact on *in vitro* biofilm formation in *S. aureus*. Whether growth of biofilms on biotic surfaces will be equally affected by Nuc remains to be determined. While it is challenging to relate *in vitro* observations to pathogenesis *in vivo*, *S. aureus* is able to persist in healthcare settings on the abiotic surfaces of fomites. There are numerous examples of MSSA and MRSA strains being isolated from hospital objects [68], [69], [70], [71], [72], [73], and MRSA has the ability to persist for periods up to 2 weeks on environmental surfaces in hospitals [74], [75]. Recent studies have demonstrated that environmental contamination may contribute to the spread of USA300 strains [76]. Whether eDNA release and modulation of eDNA levels with Nuc will impact hospital surface colonization is worthy of further exploration. With the growing recognition for the role of eDNA in bacterial biofilms, the use of nuclease enzymes as a potential means of controlling *S. aureus* attachment warrants further study.

## Materials and Methods

### Bacterial strains and growth conditions

Bacterial strains and plasmids used are described in Table 1. *E. coli* cultures were grown in Luria-Bertani (LB) broth or on LB agar, and *S. aureus* strains were grown in tryptic soy broth (TSB) or on tryptic soy agar (TSA) unless otherwise indicated. Difco methyl green DNase test agar used to examine nuclease production in *S. aureus* was purchased from BD (Sparks, MD) and prepared according to manufacturer's instructions. Plasmids in *E. coli* were maintained using antibiotic concentrations (in µg/ml) of ampicillin (Amp), 100. Plasmids in *S. aureus* were maintained using antibiotic concentrations of chloramphenicol (Cam), 10; erythromycin (Erm), 10; and tetracycline (Tet), 10, unless otherwise noted. Strains were incubated at 37°C with liquid cultures shaken at 200 RPM. When required, the growth medium was supplemented with glucose at concentrations of 0.2% or 0.4% W/V.

**Table 1:** Strains and Plasmids.

Strain or Plasmid	Description	Source or reference
Strains		
*E. coli* strains		
BW25141	Cloning strain	[88]
*S. aureus* strains		
AH845 (LAC)	USA300 CA-MRSA	[44]
AH1012	SH1000 *ΔsigB*	[12]
AH1263	USA300 CA-MRSA Erm^S^ (LAC*)	[16]
AH1292	AH1263 *Δagr*::TetM	This work
AH1483	AH1263 *ΔsigB*	This work
AH1525	AH1263 *sarA*::Kan	This work
AH1680	AH1263 *nuc*::LtrB	This work
AH1921	AH1263 *nuc*::LtrB *ΔsigB*	This work
COL	HA-MRSA	[50]
AH2496	COL *nuc*::LtrB	This work
MW2	USA400 CA-MRSA	[51]
AH2497	MW2 *nuc*::LtrB	This work
Newman	MSSA, Type 5 capsule producer	[48]
AH2495	Newman *nuc*::LtrB	This work
RN4220	Restriction deficient cloning host	[89]
SF8300	USA300 CA-MRSA	[52]
AH2498	SF8300 *nuc*::LtrB	This work
SH1000	*sigB* ^+^ derivative of NCTC8325-4	[46]
SH1001	SH1000 *Δagr*::TetM	[46]
SH1002	SH1000 *sarA*::Kan	[46]
TCH1516	USA300 CA-MRSA	[53]
AH2499	TCH1516 *nuc*::LtrB	This work
UAMS-1	Osteomyelitis isolate	[49]
UAMS-1471	UAMS-1 *nuc*	[14]
UAMS-1472	UAMS-1471 + *nuc*	[14]
UAMS-155	UAMS-1 *agr*::tet	[14]
UAMS-929	UAMS-1 *sarA*::kan	[14]
Plasmids		
pALC2073	Tet inducible expression plasmid	[79]
pALC2073-*nuc*	pALC2073 with *nuc* cloned into SacI and KpnI sites	This work
pCM20	pCE with P*_nuc_* - sGFP	This work
pCM28	pDB59 MCS	[81]
pCM28-*nuc*	pCM28 with *nuc* cloned into NheI and EcoRI sites	This work
pRMC2	Low-range Tet inducible expression plasmid	[80]
pRMC2-*nuc*	pRMC2 with *nuc* cloned into SacI and KpnI sites	This work

### Recombinant DNA and genetic techniques

Plasmid DNA was prepared from *E. coli* and electroporated into *S. aureus* RN4220 as previously described [77]. DNA was moved from RN4220 into other *S. aureus* strains through transduction with bacteriophage α80 or 11 [78]. All restriction enzymes and enzymes for DNA modification were purchased from New England Biolabs (Beverly, MA) and used according to manufacturer's instructions. Oligonucleotides were synthesized by Integrated DNA Technologies (Coralville, IA). Non-radioactive sequencing was performed at the University of Iowa DNA Sequencing Facility.

### Plasmid construction

#### Nuclease promoter fusion

The nuclease promoter region was amplified by PCR from AH1263 genomic DNA using the oligonucleotides CLM400: 5′ GTTGTTAAGCTTGTAAATTATAAGTTATACATCTCG 3′ and CLM404: 5′ GTTGTTGGTACCCTTTTTAGTTAATTTTAATATTAAACG 3′. The PCR product was purified, digested by HindIII and KpnI and ligated to pCM11 [13], also cut by the same enzymes. The plasmid was confirmed by DNA sequencing. The *nuc* promoter sGFP plasmid was designated pCM20.

#### Tetracycline-inducible vectors

The *nuc* gene was amplified by PCR from AH1263 genomic DNA using the following oligonucleotides: MRK24: 5′ GTTGTTGGTACCACTAAAAAGAAAGAGGTGTTAGTTATGACAGAATACTTATTA 3′ and MRK25: 5′ GTTGTTCTCGAGTTATTGACTGAATCAGTGTCT 3′. The PCR product was purified and digested using the restriction enzymes SacI and KpnI, then ligated to pALC2073 [79] and pRMC2 [80], also cut by SacI and KpnI. The plasmids were confirmed by DNA sequencing and called pALC2073-*nuc* and pRMC2-*nuc*, respectively.

#### Nuclease complementation plasmid

The *nuc* gene, along with 360 bp of the upstream promoter region and 100 bp downstream, was amplified by PCR from AH1263 genomic DNA using the following oligonucleotides: MRK26: 5′ GTTGTTGCTAGCGTAAATTATAAGTTATACATCTCG 3′ and MRK27: 5′ GTTGTTGAATTCAATACACTTACTTTTGATACTATTTAC 3′. The resulting PCR product was digested using the enzymes NheI and EcoRI, then ligated to pCM28 [81] cut by the same enzymes. Plasmid construction was confirmed by DNA sequencing and called pCM28-*nuc*.

### Construction of nuclease mutant

The nuclease mutant was constructed using the Targetron Gene Knockout System (Sigma, TA0100). Primers (antisense at position 532/533) as designed by the Targetron Design Site are: CLM413: 5′ AAAAAAGCTTATAATTATCCTTAAAGCTCCGTTTAGTGCGCCCAGATAGGGTG 3′, CLM414: 5′CAGATTGTACAAATGTGGTGATAACAGATAAGTCCGTTTACCTAACTTACCTTTCTTTGT 3′, CLM415: 5′ TGAACGCAAGTTTCTAATTTCGGTTAGCTTCCGATAGAGGAAAGTGTCT 3′.

PCR was performed according to the Targetron protocol. The PCR product was gel purified, digested by BsrG1 and HindIII and ligated to the *S. aureus* Targetron donor plasmid pNL9164 (Sigma, T6701) digested by the same enzymes. After electroporation into *E. coli*, the colonies were screened by PCR and subsequently sequenced. The plasmid retargeted to the nuclease gene was designated pNL-532. pNL-532 was electroporated into RN4220 and subsequently phage transduced into AH1263 at 30°C. An overnight culture grown in TSB with Erm was diluted 1∶100 in fresh media and grown at 30°C to an optical density (OD) at 600 nm of 0.5. Addition of CdCl_2_ to 10 µM followed by growth at 30°C for 90 min induced expression from the cadmium promoter. The cells were diluted and plated on TSA with Erm at 30°C. Colony PCR was used to screen for insertions using oligonucleotides CLM400: 5′ GTTGTTAAGCTTGTAAATTATAAGTT ATACATCTCG 3′ and CLM405: 5′ CAGTGACACTTTTACAATGAGC 3′. Positive colonies showed a 900 bp insertion. To cure the plasmid, an intron-positive colony was grown overnight at 42°C in TSB without antibiotic, plated and subsequently screened for erythromycin sensitive colonies. The *nuc*::LtrB mutant was designated strain AH1680.

### Identification of anti-biofilm activity by chromatography

An overnight culture of strain AH1096 (LAC Δ*sigB*) was inoculated 1∶500 into 200 ml of TSB in a 1 L flask and grown at 37°C with shaking (200 rpm) for approximately 20 hr. Cells were removed by centrifugation at 6000× g for 15 min at 4°C, and the conditioned media was filter sterilized. The media was concentrated approximately 100-fold using Amicon Ultra-15 3 K centrifugal filter units (Millipore, Bedford, MA) and dialyzed at 4°C against 10 mM sodium phosphate pH 6.5 (6×4 L) using dialysis tubing with a 3350MW cut-off. Following dialysis the conditioned media was concentrated to ∼2.5 ml using an Amicon Ultra-15 3 K centrifugal filter unit, and 2.4 ml was loaded onto a Toyopearl CM-650M (Tosoh Biosciences, Tokyo, Japan) column (1 cm×15 cm) equilibrated with 10 mM sodium phosphate pH 6.5 at 4°C. The column was washed with ∼90 ml of 10 mM sodium phosphate pH 6.5 at a flow rate of ∼1 ml/min until no protein was detected in the column effluent. Proteins were eluted using a 500 ml linear gradient of 0–0.3 M NaCl in 10 mM sodium phosphate pH 6.5 at a flow rate of ∼1 ml/min. Elution fractions (a total of 90, ∼5 ml elution fractions) were assayed for protein concentration by mixing 60 µl of the fraction with 200 µl of Bradford reagent and measuring the OD at 595 nm. The anti-biofilm activity of all fractions (the 18 flow-through and 90 elution fractions) was determined using a 96-well microtiter plate biofilm assay based on that previously described [9]. Specifically 66% TSB supplemented with 0.2% glucose was inoculated 1∶1000 with an overnight culture of SH1001 and then 190 µl aliquots of this culture were transferred to wells of 96-well microtiter plates (Corning 3596) that contained 10 µl of each fraction. 18 control wells were included in the assay that contained 10 µl of 10 mM sodium phosphate pH 6.5 and 190 µl of the SH1001 culture. Plates were incubated at 37°C with shaking (200 r.p.m.) for 15 hr and cultures were removed by gentle aspiration. Wells were washed twice with 200 µl of water, stained for 10 min with 200 µl of 0.1% crystal violet in water, and washed twice with 200 µl of water. Liquid was removed from the wells following each wash or staining step by gentle aspiration. Following the final wash, bound crystal violet was solubilized in 200 µl of 2-propanol and quantified by measuring OD at 615 nm.

### Identifying proteins by mass spectrometry

Fractions identified as having anti-biofilm activity (elution fractions 34–41 and 57–61) were analyzed by SDS-PAGE [82], and the protein bands in these fractions were cut from the gels and sent to the University of Iowa Proteomics Facility for identification by mass spectrometry. Proteins were digested with trypsin and extracted from the gels [83] and the resulting peptides were analyzed by MALDI-TOF (matrix-assisted laser desorption/ionization time-of-flight) mass spectrometry using a Bruker Biflex III instrument. The identities of the proteins was determined by submitting the mass spectrometric data for peptide mass fingerprinting using MASCOT [84].

### Development of a FRET based nuclease assay

Quantitative assays of nuclease activity typically measure the release of acid soluble oligonucleotides following nuclease digestion of DNA, with one unit of activity corresponding to a change in optical density of 1.0 at 260 nm at 37°C and pH 8.0 [85]. We developed a simple Fluorescence Resonance Energy Transfer (FRET) based assay capable of efficiently measuring the nuclease activity in conditioned media over a large dynamic range. The FRET substrate, a “PrimeTime™” qPCR probe purchased from Integrated DNA Technologies (Coralville, IA), consists of a short (15 mer) single-stranded oligonucleotide that is modified at the 5′ end with a Cy3 fluorophore and at the 3′ end with Black Hole Quencher 2 (BHQ2). The sequence of the substrate (5′ CCC CGG ATC CAC CCC 3′) is the same as that reported by Lee et al. [86], with an additional C at the 3′ end. When the oligonucleotide is intact, the Cy3 and BHQ2 are close enough that fluorescence is quenched, but when the oligonucleotide is cleaved, fluorescence from Cy3 is proportional to the amount of cleavage and can be used to quantify nuclease activity. Fluorescence measurements were made by mixing 25 µl of FRET substrate, diluted to 2 µM in buffer consisting of 20 mM Tris pH 8.0 and 10 mM CaCl_2_, with 25 µl of conditioned media (diluted with TSB as necessary) in the well of a microtiter plate (Corning), and measuring the rate of fluorescence change (excitation 552 nm/emission 580 nm) at 30°C in a Tecan Infinity 200 M plate reader. Initial reaction velocities were determined by linear least-squares fitting and converted to Units of Nuc activity per mL (U/mL) using a standard curve that was generated using various amounts of purified Nuc enzyme (0.1, 0.5, 0.025, 1.0, and 10 U/ml) purchased from Worthington Biochemicals (Lakewood, NJ). Points on the Nuc standard curve are the average of four kinetic measurements. The units of nuclease activity reported here are equivalent to reported values [85], where one unit of activity corresponds to a change in optical density of 1.0 at 260 nm at 37°C, pH 8.0, with DNA as a substrate. The detection limit for purified Nuc dissolved in growth media was found to be 0.015 U/ml, using the assay conditions and procedure employ here

### Growth dependent expression of nuc in LAC and LAC mutants

Overnight cultures of strains AH1263 (WT), AH1292 (*agr*), and AH1483 (*sigB*) grown in BHI were inoculated 1∶1000 into 200 ml of BHI or 200 ml of BHI supplemented with 0.4% glucose in 1 L flasks. Cultures were grown at 37°C with shaking (200 rpm). At designated times, approximately 5 mL of culture was removed from each flask to measure the cell density, pH, and Nuc activity with the FRET assay. Cell densities (OD at 600 nm) were measured in Thermo Spectronic Genesys20. The culture that remained after measuring cell density was filter sterilized and assayed for Nuc activity using the FRET assay described above in triplicate. The pH of the remaining sterile conditioned media was measured using an Acumet AB15 pH meter (Fisher Scientific).

### Microtiter biofilm assays for controlled nuc expression experiment

A modified microtiter assay was developed to assess biofilm formation and Nuc activity levels. Briefly, overnight cultures of strains grown in TSB supplemented with 0.2% glucose were subcultured at 1∶1000 into 66% TSB supplemented with 0.2% glucose. After growing cultures at 37°C for ∼1 hr with shaking (200 rpm), 1 mL aliquots were transferred to 24-well tissue culture treated polystyrene plates (Corning 3548). For aTET induction experiments, a total of eight wells per aTET concentration were assayed for each strain. Plates were grown at 37°C with shaking (200 rpm) for 15 hr. To measure Nuc activity in the wells, 400 µl of culture was removed and the cultures were pooled according to test condition. Bacteria were removed using 0.22 µm filters and Nuc activity was measured using the FRET assay. To quantify biofilm formation, remaining culture was removed by gentle aspiration, wells were washed three times with 1 ml of water, stained with 0.1% crystal violet for 10 min, and again washed three times with 1 ml of water. After solubilizing the bound crystal violet in 1 ml of 2-propanol, 200 µl was transferred to a 96-well plate and OD at 615 nm was measured in a plate reader. Reported relative biomass is the average of eight wells.

### Microtiter biofilm assays for nuc versus biomass correlation

Overnight cultures grown in BHI were subcultured at 1∶1000 into BHI supplemented with 0.4% glucose. After growing cultures at 37°C for ∼40 min with shaking (200 r.p.m.), 750 µl aliquots (eight wells per strain) were transferred to 24-well tissue culture treated polystyrene plates (Corning 3548). Plates were grown at 37°C with shaking (200 r.p.m.) for 15–16 hr. To measure Nuc activity in the wells, 400 µl of culture was removed from each well and the cultures were pooled according to strain. Bacteria were removed using 0.22 µm syringe tip filters and nuclease activity was measured in triplicate using the FRET assay at a substrate concentration of 2 µM. Biomass was quantified using a slightly modified staining procedure that incorporated a single washing step after cultures were removed from the wells, since the *nuc* single and *nuc sigB* double mutant biofilms were found to detach from the plates in sheets during the multiple washing steps of a standard assay. Specifically, the remaining culture was removed from the wells by gentle aspiration using a 26.5 gauge needle with the bevel turned toward the wall of the well and wells were washed with 1 ml of water and stained with 200 µl of 0.1% crystal violet for 15 sec. After removing unbound crystal violet by aspiration, bound crystal violet was solubilized in 1 ml of 2-propanol and 200 µl was transferred to a 96-well plate and OD at 615 nm was measured in a plate reader. This modified procedure was used for all of the mutant strains, not just the *nuc* and *nuc sigB* double mutants. Reported relative biomass are the average of eight wells.

### Flow cell biofilm assays

Flow cell biofilms were prepared in a similar manner as described [9] with some modifications. Briefly, *S. aureus* cultures were grown overnight in 5 mL TSB and diluted 1∶10 in sterile water. Flow cell chambers were inoculated with 1 mL of the diluted culture and bacteria were allowed to attach to acid-etched glass coverslips for 1 hr at room temperature. Laminar flow was initiated at a rate of 3.75 rpm, and the flow cells and media were incubated at 37°C. Biofilm growth media consisted of 2% TSB supplemented with 0.2% glucose, and when required, plasmids were maintained at an antibiotic concentration of 1 µg/mL. For imaging, live/dead staining was performed using the live stain SYTO9 and dead stain Toto3 or ToPro3 (as indicated) at a concentration of 1 µM in sterile PBS, as instructed in the BacLight live/dead staining kit (Invitrogen). Confocal laser scanning microscopy (CLSM) was performed on a Nikon Eclipse E600 microscope using the Radiance 2100 image capturing system (Biorad). Image acquisition was performed with the Laser Sharp 2000 software (Zeiss), and images were processed using the Volocity program (Improvision). Statistical analysis on flow cell biofilms was done using the COMSTAT program [87].

### Extracellular DNA isolation

Strains for eDNA preparations were grown overnight in TSB at 37°C shaking at 200 RPM. Fresh media was inoculated at a 1∶1000 dilution and grown for 4 hr with shaking at 37°C to an OD at 600 nm of 2.0. 5 mL of each culture was collected and filter sterilized to remove cells. Proteins were extracted from the supernatants using 1 volume of Phenol/Chloroform/Isoamyl alcohol (Roche, Indianapolis) followed by centrifugation for 15 min at 3,725× g to separate phases. DNA was precipitated by addition of 2.5 volumes 100% ethanol and 1/10 volume 3 M sodium acetate to the aqueous phase. Precipitations were incubated overnight at −20°C and spun down the following day at 22,000× g for 15 minutes, followed by a wash with cold 70% ethanol. Pellets were air dried and resuspended in TE buffer. eDNA samples were run on 1.0% agarose gels with 1 kb plus DNA ladder (Invitrogen, Carlsbad, CA). Genomic DNA used as a control for size comparison was prepared by lysis of *S. aureus* cells with Lysostaphin (Ambi Products, Purchase, NY) for 1 hr at 37°C followed by purification of chromosomal DNA with the Puregene Yeast/Bacteria Kit B (Qiagen, Maryland) according to the manufacturer's protocol. Gels were stained with ethidium bromide to visualize high MW DNA.

### DNase zymography

An overnight culture was used to inoculate 20 mL TSB at an initial OD at 600 nm of 0.1. Cultures were allowed to grow for approximately 24 hrs at 37°C with shaking at 200 rpm. Cell-free spent media was prepared by filtering through a 0.22 µM syringe filter (Millex-GS). Spent media was diluted 1∶5 in TSB and then mixed with an equal volume of SDS-PAGE sample buffer without β-mercaptoethanol. 7.5 µl was electrophoresed on a 12% SDS-PAGE that included 200 µg/mL heat-treated salmon sperm DNA (Invitrogen). To remove SDS following electrophoresis, gels were washed with 50 mL portions of 2.5% (v/v) Triton X-100 in distilled water (2 times, 10 min each), 2.5% Triton X-100 in 50 mM Tris-HCl, pH 7.4, buffer (2 times, 10 minutes each), and Tris buffer alone (2 times, 10 min each). After washing, gels were placed in 50 mL of Tris buffer and incubated at 37°C for 15 min. Gels were stained in ethidium bromide for 10 minutes, destained by rinsing 3× in distilled water, and imaged using a Gel Doc 2000 (Bio-Rad).

### Immunoblots for Nuc in spent media

*S. aureus* strains were grown overnight in TSB containing appropriate antibiotics. Overnight cultures were diluted to an OD at 600 nM of 0.1 in 5 mL TSB containing antibiotics and grown with shaking at 37°C for 15 hr. Cells were removed by centrifugation, and the supernatant was clarified using a 0.22 µm Spin-X centrifuge filter (Corning). Filtered supernatants were mixed 1∶1 with SDS-PAGE loading buffer, and 5 µL of each sample was electrophoresed on 12% SDS-PAGE. The proteins were transferred to nitrocellulose or Immobilon-P PVDF membranes (Millipore) using a Protean II device (Bio-Rad Laboratories). Membranes were blocked overnight at 4°C with 5% milk in Tris-buffered saline (20 mM Tris-HCl, pH 7.0, with 137 mM NaCl) containing 0.1% Tween 20 (TBST). Sheep anti-DNase IgG conjugated to horseradish peroxidase (Toxin Technology, Sarasota, FL) was diluted 1∶2,500 in 5% milk in TBST and incubated with the membranes at room temperature for 2 hours. Membranes were washed with agitation for 15 min, twice for 5 min with TBST, and developed with SuperSignal West Pico chemiluminescent substrate followed by exposure to X-ray film. Concentrations of protease inhibitors were as follows: 1 mM EGTA, 200 µM PMSF, and 10 µM E-64.

## Supporting Information

Figure S1 **Processing of Nuc protein.** On each panel the Nuc protein is labeled as the processed forms NucB or NucA. For panels **A** and **B**, LAC WT and strains with mutations in the *nuc* gene and *agr* and *sigB* regulators were grown 18 hr in TSB, and cells were removed by filtration. An additional culture was prepared with complemented *nuc* mutant. **A.** Immunoblot for Nuc. **B.** DNA zymography. **C.** An immunoblot of the spent media from the LAC Δ*sigB* mutant grown with protease inhibitors E64, EGTA, PMSF, or a cocktail of these three inhibitors (PIC).
(TIF)
