# Supplementary figures and images for: Nuclease Modulates Biofilm Formation in Community-Associated Methicillin-Resistant Staphylococcus aureus
Source: PLoS One. 2011 Nov 11;6(11):e26714. doi: 10.1371/journal.pone.0026714 (PMC3214024; doi:10.1371/journal.pone.0026714)

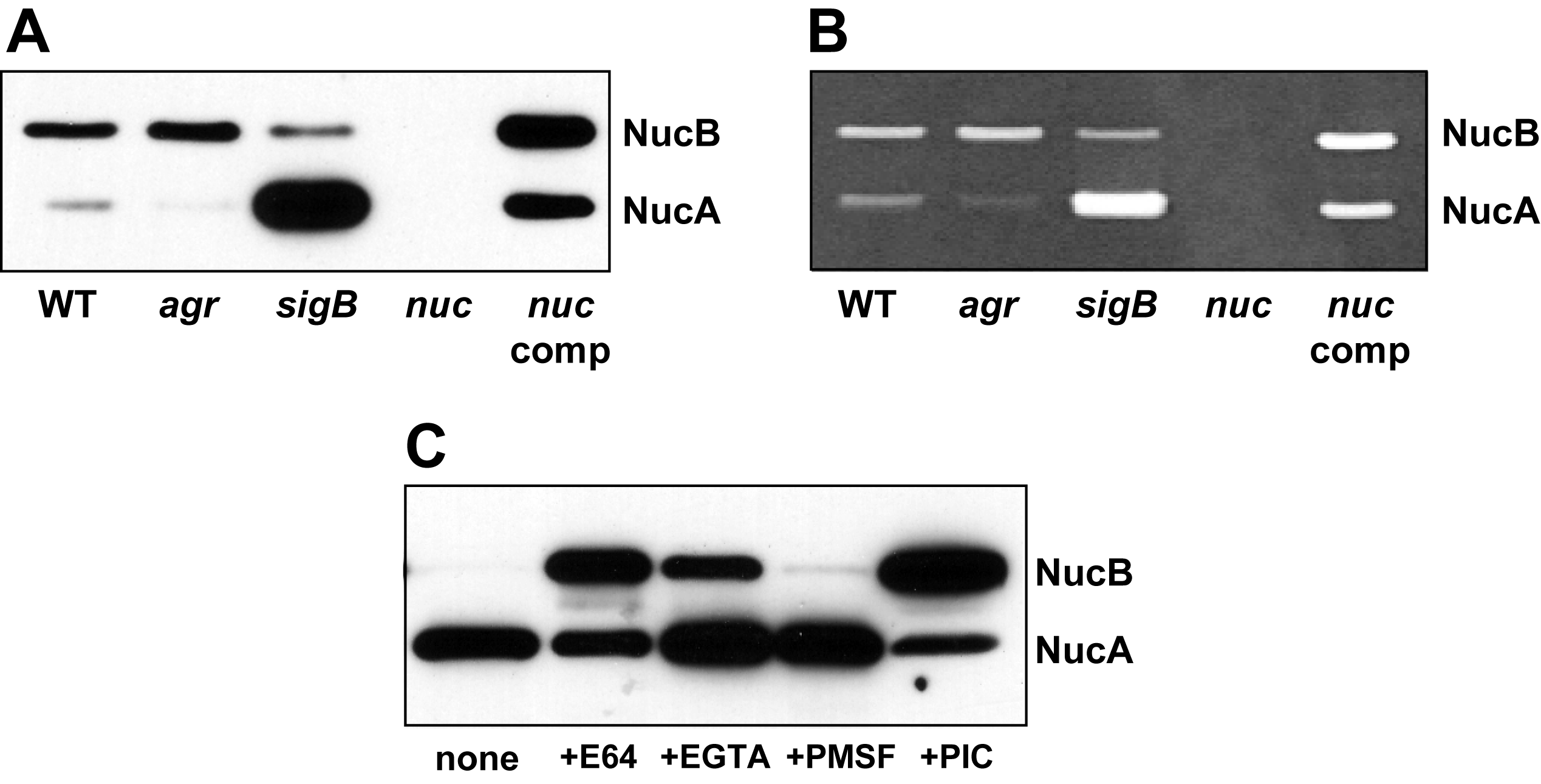

Supplement: Figure S1 — Processing of Nuc protein. On each panel the Nuc protein is labeled as the processed forms NucB or NucA. For panels A and B, LAC WT and strains with mutations in the nuc gene and agr and sigB regulators were grown 18 hr in TSB, and cells were removed by filtration. An additional culture was prepared with complemented nuc mutant. A. Immunoblot for Nuc. B. DNA zymography. C. An immunoblot of the spent media from the LAC ΔsigB mutant grown with protease inhibitors E64, EGTA, PMSF, or a cocktail of these three inhibitors (PIC). (TIF) [file pone.0026714.s001.tif]
